# Preoperative Right Ventricle–to–Pulmonary Artery Coupling Correlates With Intensive Care Length of Stay After Pulmonary Endarterectomy

**DOI:** 10.1053/j.jvca.2025.12.013

**Published:** 2025-12-19

**Authors:** Mads Dam Lyhne, Mark Stoltenberg Ellegaard, Kasper Krohn Korsholm, Jacob Valentin Hansen, Jacob Gammelgaard Schultz, Stine Andersen, Mads Jønsson Andersen, Lars Bo Ilkjær, Vasileios Zochios, Anders Morten Grejs, Peter Juhl-Olsen, Asger Andersen

**Affiliations:** * Department of Clinical Medicine, Aarhus University, Aarhus, Denmark; † Department of Anesthesiology and Intensive Care, Aarhus University Hospital, Aarhus, Denmark; ‡ Department of Cardiology, Aarhus University Hospital, Aarhus, Denmark; § Department of Cardiac and Vascular Surgery, Aarhus University Hospital, Aarhus, Denmark; ∥ Department of Critical Care Medicine and ECMO, University Hospitals of Leicester NHS Trust, Glenfield Hospital, Leicester, United Kingdom; ¶ Department of Cardiovascular Sciences, University of Leicester, Leicester, United Kingdom

**Keywords:** chronic thromboembolic pulmonary hypertension, right ventricular function, echocardiography, right heart catheterization, vasopressor

## Abstract

**Objectives:**

To investigate whether preoperative noninvasive measurement of right ventricle–to–pulmonary artery coupling, assessed by the tricuspid annular plane systolic excursion–to–pulmonary artery systolic pressure (TAPSE:PASP) ratio, is associated with perioperative outcomes following pulmonary endarterectomy (PEA).

**Design:**

Retrospective, single-center cohort study.

**Settings:**

Tertiary university hospital: national center for chronic thromboembolic pulmonary hypertension (CTEPH) treatment.

**Participants:**

Patients with CTEPH eligible for PEA.

**Interventions:**

Patients underwent transthoracic echocardiography and right heart catheterization before undergoing PEA. The TAPSE:PASP ratio was calculated from preoperative echocardiography and dichotomized at 0.17 mm/mmHg.

**Measurements and Main Results:**

The primary outcome was intensive care unit (ICU) length of stay (LOS). Secondary outcomes included vasoactive and inotropic therapy, representing perioperative hemodynamic instability. Sixty patients were included. The median TAPSE:PASP ratio was 0.20 mm/mmHg (interquartile range [IQR], 0.15-0.29 mm/mmHg); 38% of patients had ratios below 0.17 mm/mmHg. A lower TAPSE: PASP ratio was associated with higher N-terminal pro-brain natriuretic peptide levels and pulmonary vascular resistance, as well as greater right ventricular dilatation. The median ICU LOS was longer in patients with a TAPSE:PASP ratio below 0.17 mm/mmHg (96 hours [IQR, 60-218 hours] *v* 63 hours [IQR, 41-93 hours], p = 0.0044), with an odds ratio (OR) of 3.45 (95% confidence interval [CI], 1.16-11.11) for ICU LOS greater than 72 hours. Patients with lower TAPSE:PASP ratios had a higher (OR, 3.33; 95% CI, 1.11-10.00; p = 0.032) and prolonged (OR, 3.70; 95% CI, 1.23-11.11; p = 0.019) need for vasoactive and inotropic therapy.

**Conclusions:**

A lower preoperative TAPSE:PASP ratio was associated with prolonged ICU stay and hemodynamic compromise after PEA. The TAPSE:PASP ratio may serve as a valuable, noninvasive tool for preoperative risk stratification in patients with CTEPH undergoing surgical treatment.

PULMONARY ENDARTERECTOMY (PEA) is the definitive treatment for chronic thromboembolic pulmonary hypertension (CTEPH), although it entails a highly complex anesthetic, surgical, and postoperative course.^1–4^ Despite low perioperative mortality rates in high-volume centers, complications remain common and include hemodynamic instability due to, for example, systemic inflammation, bleeding, residual pulmonary hypertension, or right ventricular failure that may prolong intensive care unit (ICU) stays.^3,5^ ICU length of stay (LOS) predicts mortality,^6^ which underscores the importance of accurate risk stratification and careful patient selection.^7,8^

Different prognostic markers—including brain natriuretic peptide, coagulation biomarkers, central venous oxygen saturation, and pulmonary arterial hypertension (PAH) risk scores—have been explored.^9–12^ However, these may not fully reflect the pathophysiology of CTEPH with elevated pulmonary pressures and right ventricular dysfunction and failure.^13–15^ Under healthy conditions, the right ventricle (RV) and pulmonary artery (PA) are mechano-energetically well balanced in their low-pressure system, that is, normal RV-PA coupling. As the pulmonary pressure increases in CTEPH, RV function needs to adapt to maintain RV-PA coupling, whereas uncoupling occurs when RV adaptation is insufficient to meet the functional demand.^8,16^ Incorporating RV-PA coupling metrics into CTEPH risk stratification may offer additional prognostic value.

The tricuspid annular plane systolic excursion–to–pulmonary artery systolic pressure (TAPSE:PASP) ratio is an emerging echocardiographic metric that couples RV function and pulmonary arterial pressure. It correlates with invasive pulmonary hemodynamics and improves following mechanical interventions such as PEA.^17–20^ Yet, its role as a predictor of perioperative morbidity remains unclear. This study aimed to investigate the association between the TAPSE:PASP ratio and perioperative outcomes in CTEPH patients undergoing PEA. The authors hypothesized that a lower TAPSE:PASP ratio would be associated with prolonged ICU stay and increased vasopressor requirements.

## Methods

### Design and Ethics

The authors conducted a retrospective, observational cohort study investigating CTEPH patients undergoing PEA at a single center from September 2015 to August 2021. All procedures were performed in compliance with relevant laws and international guidelines. The local ethical committee approved the study (No. 1-16-5-72-719-23). The requirement for informed consent was waived given the study’s retrospective and non-experimental design, but the privacy rights of human subjects were observed. Data are reported in accordance with the Strengthening the Reporting of Observational Studies in Epidemiology (STROBE) guidelines^21^; the checklist can be found in Appendix A.

### Eligibility Criteria

Inclusion criteria were Danish patients aged 18 years or older with diagnosed CTEPH according to guidelines^14^ after multidisciplinary evaluation undergoing PEA at the authors’ institution during the study period. Patients were excluded if data on key predictor variables or primary or secondary outcomes were missing.

### Outcomes

The primary outcome was ICU LOS, typically expected to be 3 days postoperatively but often extending to 4 to 15 days.^3,4^ A 24-hour difference was defined a priori as clinically significant. The authors hypothesized that patients with a low TAPSE:PASP ratio would have a prolonged ICU stay. The secondary outcomes were peak administrations,^22^ duration, and accumulated dose^23^ of vasoactive and inotropic agents administered perioperatively and during ICU stays, used as a surrogate for hemodynamic instability.

### Preoperative Data

Data from transthoracic echocardiography (TTE) performed during the diagnostic workup (71 days before PEA [interquartile range (IQR), 49-101 days before PEA]) were analyzed as previously described.^18^ The authors extracted information on tricuspid annular plane systolic excursion (TAPSE), pulmonary artery systolic pressure (PASP), tricuspid regurgitation gradient, RV basal diameter, right atrial area, left ventricular (LV) ejection fraction, and LV diameter. From these data, the TAPSE:PASP and RV:LV diameter ratios were calculated.

Right heart catheterization (RHC) data included mean PA pressure, PA wedge pressure, pulmonary vascular resistance (PVR), central venous pressure, cardiac output or index, and mixed venous oxygen saturation. PA pulsatility index was calculated as pulmonary pulse pressure divided by mean right atrial pressure.^24^ Additional preoperative metrics included 6-minute walk distance, Borg dyspnea scale, New York Heart Association class, N-terminal probrain natriuretic peptide (NT-proBNP) level, creatinine level, and European System for Cardiac Operative Risk Evaluation (EuroSCORE) II.^25^

### Perioperative Data

Patient records were reviewed for intraoperative and postoperative variables including systemic and pulmonary hemodynamics, anesthetic agents, biomarkers, durations of extracorporeal circulation and deep hypothermic circulatory arrest, vasoactive medications, complications, ICU and hospital LOS, and 30- and 90-day mortality. All patients were monitored using PA catheters (continuous cardiac output and mixed venous oxygen saturation) during surgery and in the ICU to guide postoperative management. Catheters were removed once patients were hemodynamically stable and weaned off nitric oxide. Total vasopressor need was calculated as the accumulated norepinephrine-equivalent dose using a recently updated conversion formula incorporating total norepinephrine, epinephrine, phenylephrine, vasopressin, and methylene blue therapy.^23^ Similarly, the vasoactive-inotropic score was calculated as the highest dose of vasoactive and inotropic agents administered during the first 24 postoperative hours, including dobutamine, milrinone, norepinephrine, epinephrine, and vasopressin.^22^

### Statistics

No post hoc power calculation was performed given the fixed sample size. Data are presented as median (IQR). Patients were stratified by TAPSE:PASP tertiles, and trend tests were conducted across groups. A TAPSE:PASP ratio below 0.17 mm/mmHg, based on prior CTEPH literature,^17^ was used to dichotomize the cohort. Group comparisons used the Wilcoxon rank sum test. ICU LOS was both assessed as a continuous outcome and dichotomized at 72 hours, considered an appropriate postoperative duration.^3^ Logistic regression was used to assess associations between TAPSE:PASP ratio and outcomes (ICU LOS, hospital LOS, and circulatory support), with odds ratios (ORs) and 95% confidence intervals (CIs) reported. Comparative analyses were repeated using PVR dichotomized at the median. For discriminative capabilities, the authors performed receiver operating characteristic curve analysis and calculated the area under the curve (AUC). STATA, version 18 (StataCorp, College Station, TX), was used for all analyses. A p value < 0.05 was considered statistically significant.

## Results

**Fig 1.**
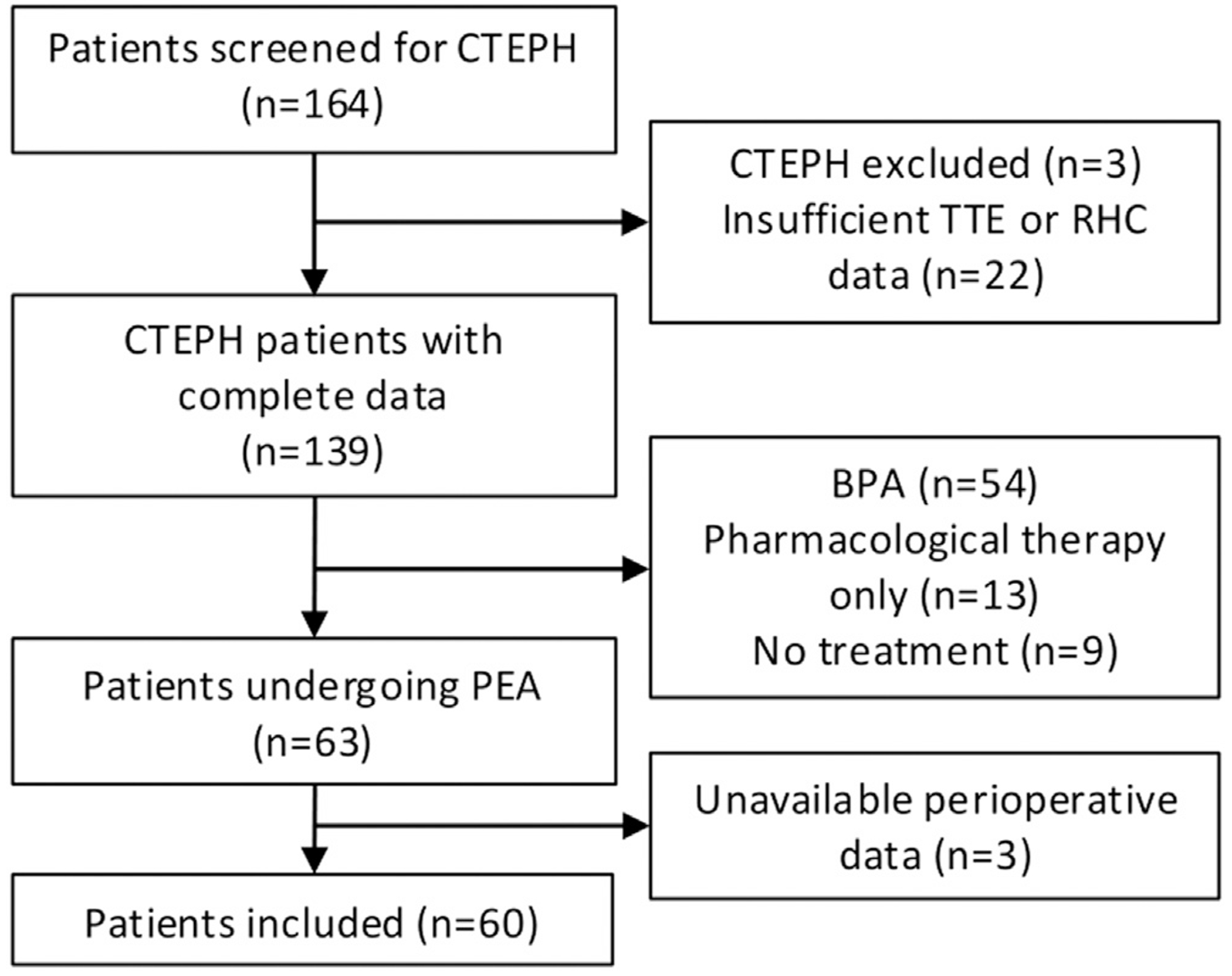
Patient selection. The patient selection process is performed according to Strengthening the Reporting of Observational Studies in Epidemiology (STROBE) guidelines. CTEPH, chronic thromboembolic pulmonary hypertension; TTE, transthoracic echocardiography; RHC, right heart catheterization; BPA, balloon pulmonary angioplasty; PEA, pulmonary endarterectomy.

A total of 164 patients with suspected CTEPH during the aforementioned period were screened, and 60 patients who underwent PEA were included in the analysis. Figure 1 presents a flowchart of the patient selection process.

### Baseline Characteristics

**Fig 2.**
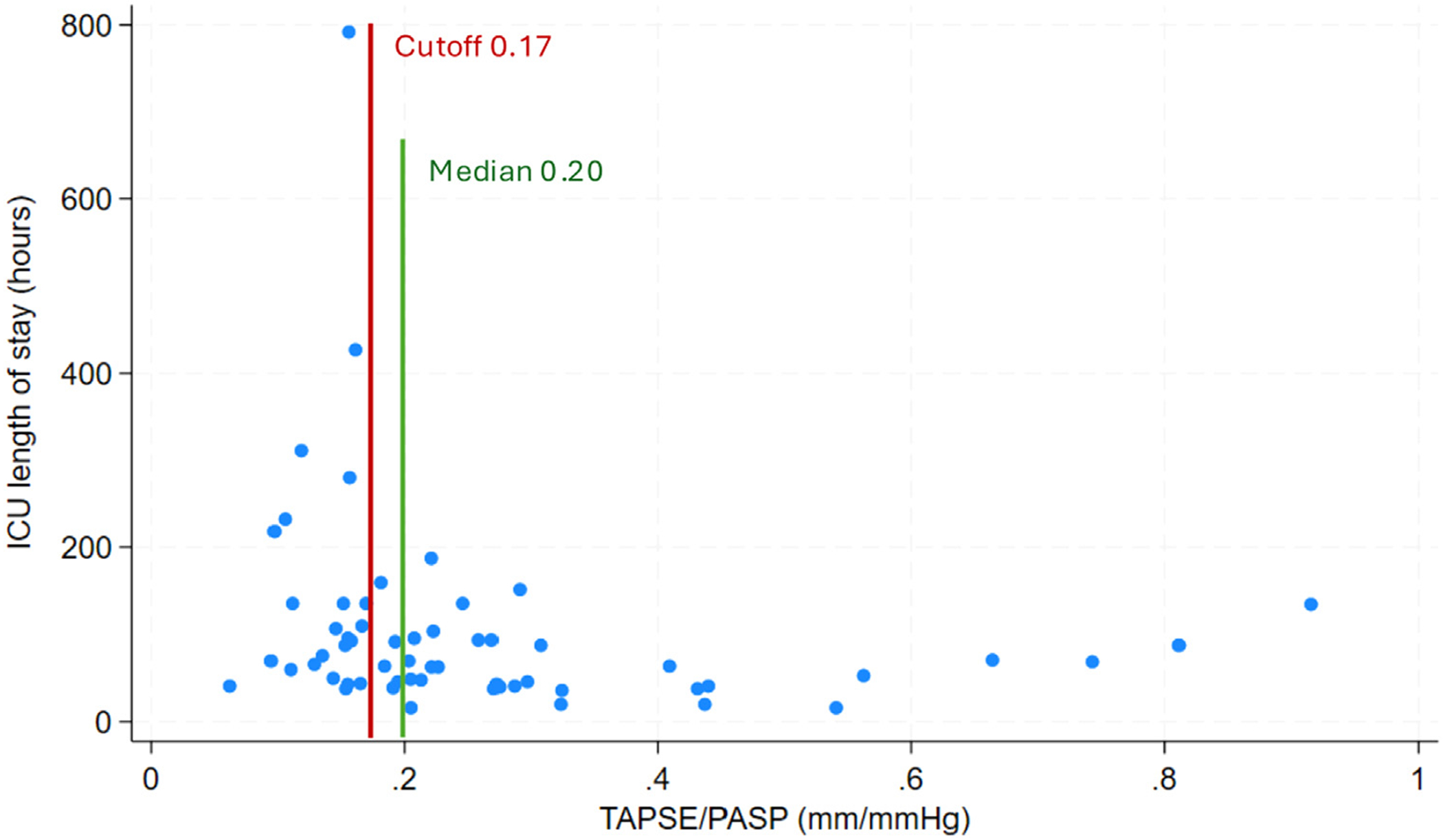
Scatter plot of preoperative tricuspid annular plane systolic excursion–to–pulmonary artery systolic pressure (TAPSE:PASP) ratio and primary outcome: intensive care unit (ICU) length of stay. One extreme outlier is excluded from the scatter plot. Patients with a low TAPSE:PASP ratio had a higher odds ratio for an increased ICU length of stay, as detailed in the “Results” section.

Baseline data are shown in Table 1, and detailed preoperative symptoms, biomarkers, echocardiographic findings, and invasive hemodynamic parameters are summarized in Table 2. The median TAPSE:PASP ratio was 0.20 mm/mmHg (IQR, 0.15-0.29 mm/mmHg), with 23 patients (38%) falling below the predefined cutoff of 0.17 mm/mmHg (Fig 2). Patients with a TAPSE:PASP ratio below the median had higher NT-proBNP levels and worse invasive hemodynamics and echocardiographic findings (Appendix Table 1). When patients were stratified into tertiles (0.06-0.15, 0.16-0.26, and 0.27-0.92 mm/mmHg), a lower TAPSE:PASP ratio was associated with higher NT-proBNP and creatinine levels, elevated pulmonary pressures and PVR, reduced cardiac index, and increased RV dilatation (Table 3).

### Anesthesia and Surgery

For induction of anesthesia, 58 of patients (97%) received midazolam; 20 (33%), propofol; 48 (80%), ketamine; 59 (98%), sufentanil; and 1 (2%), fentanyl. All received neuromuscular blockade. General anesthesia was maintained with propofol and sufentanil infusions. The median durations of extracorporeal circulation, aortic clamping, and deep hypothermic circulatory arrest were 244 minutes (IQR, 228-271 minutes), 124 minutes (IQR, 108-146 minutes), and 25 minutes (IQR, 19-36 minutes), respectively.

### Postoperative Care in ICU

All patients were transferred to the ICU postoperatively. The median intubation time was 29 hours (IQR, 26–47 hours), which differed among TAPSE:PASP tertiles. The median ICU LOS was 70 hours (IQR, 43–110 hours) (with 1 extreme outlier with a markedly prolonged ICU stay of 95 days because of a complicated, ultimately fatal course). Patients with ICU LOS above the median had a higher New York Heart Association class and European System for Cardiac Operative Risk Evaluation (EuroSCORE) II, higher pulmonary pressures and PVR, and greater vasopressor need (Appendix Table 2), whereas circulatory arrest time was not associated with ICU LOS (data not shown).

ICU LOS significantly decreased across TAPSE:PASP tertiles (95 hours [IQR, 63-225 hours], 81 hours [IQR, 48-123 hours], and 45 hours [IQR, 38-80 hours]; p = 0.004). When stratified by TAPSE:PASP ratio below 0.17 mm/mmHg, these patients had significantly longer ICU stays (96 hours [IQR, 60-218 hours] *v* 63 hours [IQR, 41-93 hours]; p = 0.004), corresponding to an OR of 3.45 (95% CI, 1.16-11.11; p = 0.026) for ICU LOS greater than 72 hours (Fig 2). The TAPSE:PASP ratio demonstrated a good ability to discriminate between patients with prolonged ICU LOS and those without (AUC, 0.65 [95% CI, 0.50-0.79]). For comparison, patients with PVR above the median had an OR of 4.03 (95% CI, 1.37-11.84; p = 0.011) for prolonged ICU stay with an AUC of 0.70 (95% CI, 0.56-0.83). An association with prolonged ICU LOS also was present for TAPSE alone (OR, 3.13 [95% CI, 1.06-9.09]; p = 0.037; AUC, 0.59 [95% CI, 0.44-0.74]) and PASP alone (OR, 3.00 [95% CI, 1.05-8.60]; p = 0.041; AUC, 0.66 [95% CI, 0.51-0.80]).

### Circulatory Support

For perioperative circulatory support, 59 of patients (98%) received norepinephrine; 2 (3%), epinephrine; 49 (82%), dobutamine; 7 (12%), milrinone; 2 (3%), vasopressin; and 1 (2%), methylene blue. For the peak administration of circulatory support, the median vasoactive-inotropic score was 10.00 (IQR, 5.25-21.45) with a significant difference across TAPSE: PASP tertiles (p = 0.019) (Table 3). Patients with a TAPSE: PASP ratio below 0.17 mm/mmHg were more likely to have a vasoactive-inotropic score above the median (OR, 3.33; 95% CI, 1.11-10.00; p = 0.032).

For the cumulative administration of circulatory support, the median accumulated norepinephrine-equivalent dose was 5,648 μg (IQR, 2,356-17,352 μg). Although higher vasopressor requirements were noted across TAPSE:PASP tertiles, the trend was not statistically significant (p = 0.11) (Table 3). Patients with a TAPSE:PASP ratio below 0.17 mm/mmHg trended toward higher accumulated vasopressor need (10,576 μg [IQR, 2,487-32,904 μg] *v* 4,292 μg [IQR, 1,974-8,516 μg]; p = 0.066), with an OR of 2.78 (95% CI, 0.93-8.33; p = 0.066) for vasopressor need above the median. PVR above the median was associated with increased vasopressor need (OR, 2.98; 95% CI, 1.04-8.53; p = 0.041). For the 49 patients (82%) receiving dobutamine, the median accumulated dose was 183 μg (IQR, 13-403 μg), with a trend toward higher usage in patients with low TAPSE:PASP ratios (346 μg [IQR, 81-749 μg] *v* 127 μg [IQR, 13-358 μg]; p = 0.086).

Regarding the duration of circulatory support, the median duration of postoperative circulatory support was 37 hours (IQR, 22-70 hours), and a significant difference was observed across TAPSE:PASP tertiles (p = 0.005) (Table 3). When patients were stratified by TAPSE:PASP ratio below 0.17 mm/mmHg, the authors found an OR of 3.70 (95% CI, 1.23-11.11; p = 0.019) for circulatory support duration above the median. For the 17 patients (28%) with data available on the duration of postoperative inhaled nitric oxide (20 ppm), the median time was 25 hours (IQR, 16-34 hours), with no significant difference across TAPSE:PASP tertiles (Table 3). The TAPSE:PASP ratio and PVR showed similar discriminative capabilities regarding need for increased or prolonged vasoactive therapy whereas TAPSE and PASP had numerically smaller AUCs (data not shown).

### Hospital Stay and Mortality

The median hospital LOS was 10 days (IQR, 8-14 days) and did not differ significantly among TAPSE:PASP tertiles or when stratified by TAPSE:PASP ratio below 0.17 mm/mmHg. Similarly, PVR was not associated with hospital LOS (data not shown).

Radiography-verified pulmonary reperfusion edema developed in 3 patients (5%), and 2 patients (3%) died during hospitalization. Eight patients (13%) were readmitted within 90 days postoperatively. No deaths occurred within 30 days postoperatively, and 1 additional patient died within 90 days postoperatively.

## Discussion

In this retrospective study, the authors found that a lower preoperative TAPSE:PASP ratio—an echocardiographic surrogate for RV-PA coupling—was independently associated with prolonged ICU LOS following PEA in patients with CTEPH. Additionally, patients with lower TAPSE:PASP ratios exhibited a higher and prolonged need for vasopressor support, indicating greater perioperative hemodynamic instability. These results are based on a cohort of well-characterized patients with detailed perioperative data. PEA multidisciplinary teams should be aware of low TAPSE:PASP ratios when deciding to offer patients PEA procedures (*v* less invasive therapeutic strategies) and include an expected prolonged ICU LOS in the planning of the perioperative period. The current investigation cannot directly prescribe how to approach patients with RV-PA uncoupling (ventilation strategy, fluid balance, RV support, pacing, etc) but may inform future research toward protecting the RV and improving postoperative outcomes and costs in this high-risk patient population.

### Invasive Versus Noninvasive Risk Stratification

Risk profiling of patients undergoing PEA surgery is an area of active interest but is complex, with both patient- and procedure-related factors influencing the outcome.^6,26^ The findings of the current study suggest that the TAPSE:PASP ratio may be a useful and accessible metric for preoperative risk stratification in candidates for PEA. The need for noninvasive surrogates in patients with PEA is debatable, however, given that preoperative RHC with direct, invasive hemodynamic investigation is available in PEA patients.^27^ Nevertheless, noninvasive strategies are usually of interest, being less expensive, safer, and easily repeatable, and the authors have previously shown an excellent correlation between TTE and RHC in the CTEPH population.^18^ Besides pressure estimates, TTE offers additional information on RV function, and TTE enables longitudinal assessment postoperatively whereas RHC is only available in a time-limited phase. The latter is especially relevant in patients with preoperative RV-PA uncoupling and in cases of residual pulmonary hypertension (PH) or postoperative RV injury. Accordingly, invasive and noninvasive strategies should not be considered competing, but rather complementary, modalities within a multimodal monitoring framework in the perioperative period.

### TAPSE:PASP ratio in CTEPH

CTEPH is characterized by pulmonary vascular obstruction and small-vessel vasculopathy.^28,29^ Pulmonary vascular flow impairment increases PVR, pulmonary arterial pressure, and RV afterload, which impairs RV function and, over time, causes RV remodeling with initial adaptation. With disease advancement, the RV progresses into RV maladaptation and failure.^8,30^ RV-PA coupling describes this relationship between RV function and its afterload and is measured by invasive pressure-volume loop recordings as the Ees:Ea ratio, in which Ees is end-systolic elastance (contractility) and Ea is arterial elastance (afterload).^16^ However, as pressure-volume loop recordings are rarely clinically available, there is a need for an accessible surrogate marker.

The TAPSE:PASP ratio has emerged as a noninvasive surrogate for RV-PA coupling and correlates well with hemodynamic measures and clinical outcomes across multiple cardiopulmonary conditions, including PAH, acute pulmonary embolism, and CTEPH.^17,18,31,32^ The ratio has been validated against invasive gold-standard methods in PAH.^16,33^ Notably, the TAPSE:PASP ratio has been shown to improve after mechanical interventions for CTEPH such as PEA^17–20^ even though TAPSE alone often decreases postoperatively due to altered pericardial tethering following sternotomy and pericardiotomy—the latter reducing interventricular dependence and thus septal position, potentially reducing RV function.^34^ In the present study, the TAPSE:PASP ratio did not perform significantly better than TAPSE or PASP individually, which has otherwise been shown previously in acute pulmonary embolism.^35,36^ This discrepancy may be explained by differences in pathophysiological development: A healthy RV cannot adapt sufficiently to an acutely elevated afterload in acute pulmonary embolism, whereas a floor effect is reached for longitudinal shortening in the slowly progressive trajectory of diseases such as PAH and CTEPH.^37^

### ICU Admission After PEA

PEA is a technically demanding procedure requiring deep hypothermia and circulatory arrest, necessitating postoperative ICU care to monitor for complications such as RV failure and reperfusion pulmonary edema.^3,7,8,38^ Anesthesia in patients with PH and RV dysfunction carries a high risk because of the hemodynamic impact of sedatives and positive pressure ventilation.^39–41^ ICU LOS predicts in-hospital mortality following PEA^6^ and is an endpoint of clinical relevance.

In the present cohort, ICU LOS was comparable to or shorter than that reported in previous PEA studies.^3,42–46^ Prior studies have identified factors such as elevated RV:LV ratio and NT-proBNP level, but not age or inflammation, as predictors of prolonged ICU stay.^11,43–46^ Low preoperative arterial oxygenation level and elevated NT-proBNP level have been associated with a higher incidence of post-PEA complications, which may warrant prolonged ICU LOS, although this was not reported.^10,44,47^ While biomarkers and computed tomography–derived metrics provide indirect assessments of RV dysfunction, the TAPSE:PASP ratio directly captures RV-PA coupling and therefore may be a more sensitive marker of perioperative risk.

In their analysis, the authors observed a clear association between a low TAPSE:PASP ratio and prolonged ICU LOS, comparable to recent findings.^48^ This was explained by more labile circulation immediately after PEA as evidenced by a higher vasoactive-inotropic score and a significantly prolonged need for vasopressor therapy. The longer ICU stay may therefore reflect that patients with impaired RV-PA coupling exhibit limited cardiovascular reserve, requiring more cautious postoperative management, extended monitoring, and slower weaning from vasoactive therapy. Moreover, time from extubation to discharge varies as ICU discharge criteria often incorporate multiple clinical factors—such as patient-related factors including respiratory function, imaging findings, diuresis, and fluid balance—that may be indirectly influenced by RV dysfunction. ICU discharge timing is additionally influenced by organizational factors such as staffing, daytime discharge routines, and ward capacity.

When patients were stratified by TAPSE:PASP ratio, the authors observed a clinically relevant median ICU LOS difference exceeding 24 hours, comparable to observations in coronary and valve surgery.^48^ This finding is of practical importance for ICU resource planning and supports integrating the TAPSE:PASP ratio into preoperative assessment algorithms. It is interesting to note that the strength of association between TAPSE:PASP ratio and ICU LOS was comparable to that of PVR, a cornerstone parameter in PH and CTEPH.^14^ Unlike invasive hemodynamics, the TAPSE:PASP ratio is safe and inexpensive, making it a feasible tool for routine use.

### Circulatory Support

The authors investigated both the peak administration rates and the durations and accumulated doses of circulatory agents. The accumulated norepinephrine-equivalent dose was used as a proxy for hemodynamic instability, offering insight beyond transient measures such as mean arterial pressure, which can be influenced by vasopressor therapy itself.^23^ While vasopressors are routinely used in PEA to maintain perfusion, they also may exacerbate RV dysfunction by increasing PVR.^1,7^ Moreover, few of the agents used targeted specific RV support; most were merely general vasopressors or inotropes. Specific agents for RV support in PEA surgery should be investigated in future studies. Prior studies in PH patients undergoing noncardiac surgery have shown that higher intraoperative vasopressor requirements correlate with worse outcomes.^49^ Although vasopressor use in the context of PEA remains underexplored, inflammatory biomarkers have been linked to both hemodynamic deterioration and prolonged vasopressor need.^44,50^ Consistent with these data, the current results suggest that patients with lower TAPSE:PASP ratios required higher peak doses of vasoactive and inotropic agents for longer durations. The current results are in line with recently published data describing the association between a low TAPSE: PASP ratio and the development of systemic hypotension and the need for vasopressor support on induction of general anesthesia in patients anesthetized for non-cardiac surgery.^51^ Accordingly, a low TAPSE:PASP ratio identifies a clinically vulnerable population.

### Limitations

The retrospective, single-center design and limited sample size of this study introduce potential selection bias and limit generalizability, given that only surgically treated CTEPH patients with complete preoperative data were included. To counter this, the authors used a TAPSE:PASP ratio cutoff derived from an independent CTEPH cohort^17^ to enhance external validity. Because of limited power, the authors were not able to investigate associations between the TAPSE:PASP ratio and long-term outcomes or rare events. Incomplete datasets induce attrition bias, but the authors reanalyzed all preoperative transthoracic echocardiograms to minimize this.^18^ In addition, while the TAPSE:PASP ratio is correlated with invasive RV-PA coupling, it is not a direct measure and is susceptible to operator variability,^8,33^ which may have introduced random error, likely biasing results toward the null. However, measurements of pulmonary arterial elastance are prone to similar methodologic limitations, and the TAPSE: PASP ratio does correlate with PVR. RV-PA coupling in this study was estimated during stable conditions prior to surgery, whereas pulmonary arterial elastance and hence RV-PA coupling are dominated by PVR and downstream pressures (eg, left atrial pressure or alveolar pressure), which change significantly during the perioperative course. Furthermore, unmeasured confounders such as comorbidities, surgical complexity, or perioperative management strategies could have influenced ICU outcomes, possibly inflating or attenuating the true association between the TAPSE:PASP ratio and clinical endpoints. Finally, as discussed earlier, the utility of noninvasive surrogates may be challenged by the availability of direct, invasive hemodynamic assessment in the PEA population.

## Conclusion

In patients undergoing PEA for CTEPH, a lower preoperative TAPSE:PASP ratio was associated with prolonged ICU stay and longer vasopressor requirements. These findings suggest that the TAPSE:PASP ratio, a noninvasive measure of RV-PA coupling, may help identify patients at higher perioperative risk. Given its accessibility and clinical relevance, the TAPSE:PASP ratio could be incorporated into routine preoperative risk assessment. Prospective studies are warranted to validate these findings and explore their impact on perioperative management strategies.

## Supplementary Material

1

2

3

Supplementary material associated with this article can be found in the online version at doi:10.1053/j.jvca.2025.12.013.

## Figures and Tables

**Table 1:** Patient Characteristics

	Data
Age, y	64 (51-72)
Male sex	35 (58)
BMI	27.2 (25.5-1.8)
Smoking	
Never	26 (43)
Past	29 (48)
Active	5 (8)
Pulmonary disease	15 (25)
Asthma	4(7)
COPD	6 (10)
Emphysema	1 (2)
Fibrosis	2 (3)
Sarcoidosis	2 (3)
Other	2 (3)
Ischemic heart disease	6 (10)
Heart failure	0 (0)
Valvular heart disease	10 (17)
Diabetes	5 (9)
Stroke	4 (7)
Thrombophilia	10 (17)
Previous pulmonary embolism	55 (92)
Preoperative treatment	
PDE-5 inhibitor	4 (7)
Antihypertensive	3 (5)
Warfarin	51 (85)
LMWH	1 (2)
DOAC	7 (12)
ASA	1 (2)
Long-term oxygen therapy	4 (7)

NOTE. Characteristics of included patients (N = 60) are presented as median (interquartile range) or number (percentage) as appropriate.

Abbreviations: ASA, acetylsalicylic acid; BMI, body mass index; COPD, chronic obstructive pulmonary disease; DOAC, direct oral anticoagulant; LMWH, low-molecular-weight heparin; PDE-5, phosphodiesterase 5.

**Table 2:** Symptoms and Patient Measurements

	Data
6MWD, m	395 (303-480)
Borg scale	5 (3-7)
NYHA class	
I	1 (2)
II	11 (19)
III	44 (76)
IV	2 (3)
NT-proBNP level, ng/L	1,231 (422-3,961)
Creatinine level, μmol/L	89 (77-103)
EuroSCORE II	1.45 (1.15-2.20)
Right heart catheterization	
Mean right atrial pressure, mmHg	9 (6-11)
Pulmonary artery systolic pressure, mmHg	84 (70-93)
Pulmonary artery diastolic pressure, mmHg	29 (22-35)
Pulmonary artery mean pressure, mmHg	48 (41-55)
Pulmonary artery wedge pressure, mmHg	10 (8-13)
Cardiac output, L/min	4.2 (3.6-5.1)
Cardiac index, L/min/m^2^	2.1 (1.7-2.4)
PVR, WU	9.1 (6.2-11.9)
PAPi	6.3 (3.9-8.6)
SvO_2_, %	57 (53-64)
Arterial oxygen saturation, %	91 (88-93)
Echocardiography	
RA area, cm^2^	23.9 (19.4-31.4)
RV diameter, mm	51 (45-57)
RV:LV ratio	1.19 (0.94-1.48)
TAPSE, mm	18 (13-20)
PASP, mmHg	82 (67-97)
TAPSE:PASP ratio, mm/mmHg	0.20 (0.15-0.29)
LV ejection fraction, %	60 (57-62)

NOTE. Symptoms, functional, biochemical, invasive, and echocardiographic characteristics of included patients (N = 60) are presented as median (interquartile range) or number (percentage) as appropriate.

Abbreviations: 6MWD, 6-minute walk distance; EuroSCORE, European System for Cardiac Operative Risk Evaluation; LV, left ventricular; NT- proBNP, N-terminal pro-brain natriuretic peptide; NYHA, New York Heart Association; PAPi, pulmonary artery pulsatility index; PASP, pulmonary artery systolic pressure; PVR, pulmonary vascular resistance; RA, right atrial; RV, right ventricular; SvO_2_, mixed venous oxygen saturation; TAPSE, tricuspid annular plane systolic excursion; WU, Wood unit.

**Table 3:** TAPSE:PASP Tertile Stratification

	TAPSE:PASP Tertile 1	TAPSE:PASP Tertile 2	TAPSE:PASP Tertile 3	p Value for Trend
6MWD, m	365 (272-456)	360 (295-498)	420 (360-470)	0.44
NT-proBNP level, ng/L	3,820 (1,763-5,188)	1,410 (640-3,470)	230 (116-568)	<0.001
Creatinine level, mmol/L	98 (81-108)	91 (80-107)	80 (66-92)	0.023
EuroSCORE II	1.61 (1.19-2.50)	1.39 (1.18-2.11)	1.17 (0.95-2.24)	0.24
Right heart catheterization				
Mean right atrial pressure, mmHg	10 (9-16)	8 (5-10)	7 (5-10)	0.011
Pulmonary artery systolic pressure, mmHg	90 (75-94)	89 (79-102)	66 (47-83)	<0.001
Pulmonary artery diastolic pressure, mmHg	34 (30-37)	29 (21-34)	22 (17-29)	<0.001
Pulmonary artery mean pressure, mmHg	52 (47-56)	50 (45-58)	39 (30-47)	<0.001
Pulmonary artery wedge pressure, mmHg	10 (8-14)	10 (7-12)	11 (9-14)	0.27
Pulmonary artery pulsatility index	4.7 (3.3-6.6)	7.5 (5.8-13.3)	5.8 (3.9-8.1)	0.011
Cardiac output, L/min	3.8 (3.2-4.1)	4.4 (3.5-5.1)	4.8 (4.2-5.4)	0.003
Cardiac index, L/min/m^2^	1.9 (1.6-2.1)	2.3 (1.7-2.5)	2.3 (2.1-2.9)	0.003
PVR, WU	11.2 (9.7-14.2)	9.6 (7.0-13.6)	5.6 (3.7-6.9)	<0.001
SvO_2_, %	54 (50-56)	57 (54-61)	65 (59-69)	<0.001
Arterial saturation, %	90 (86-92)	90 (88-93)	93 (90-95)	0.22
Echocardiography RA area, cm^2^	28.5 (23.6-33.8)	21.9 (18.3-30.3)	22 (18.5-26.2)	0.039
RV diameter, mm	53 (49-60)	49 (42-56)	46 (42-56)	0.078
RV:LV ratio	1.46 (1.15-1.67)	1.20 (0.87-1.44)	1.02 (0.90-1.19)	0.005
TAPSE, mm	13 (10-15)	18 (16-20)	22 (19-26)	<0.001
TAPSE:PASP ratio, mm/mmHg	0.14 (0.11-0.15)	0.20 (0.19-0.22)	0.37 (0.29-0.55)	<0.001
LV ejection fraction, %	61 (54-62)	59 (55-65)	60 (57-62)	0.94
In-hospital treatment				
ICU LOS, h	95 (63-225)	81 (49-123)	45 (38-80)	0.004
Intubation duration, h	33 (28-64)	29 (27-34)	27 (24-30)	0.034
Vasopressor duration, h	54 (32-169)	39 (24-64)	24 (18-41)	0.005
Inhaled nitric oxide duration (n = 17), h	25 (16-34)	30 (17-41)	10 (10-10)	0.42
Vasoactive-inotropic score	13 (7-40)	14 (6-21)	7 (4-10)	0.019
Accumulated norepinephrine-equivalent dose, μg	8,620 (2,888-36,020)	6,810 (1,942-24,612)	3,988 (2,214-6,632)	0.11
Accumulated dobutamine dose, μg	283 (42-702)	314 (90-540)	66 (0-230)	0.029

NOTE. Functional, biochemical, invasive and echocardiographic characteristics and in-hospital management data of all patients stratified by TAPSE:PASP tertiles (N = 60) are presented as median (interquartile range).

Abbreviations: 6MWD, 6-minute walk distance; EuroSCORE, European System for Cardiac Operative Risk Evaluation; ICU, intensive care unit; LOS, length of stay; LV, left ventricular; NT-proBNP, N-terminal pro-brain natriuretic peptide; PASP, pulmonary artery systolic pressure; PVR, pulmonary vascular resistance; RA, right atrial; RV, right ventricular; SvO_2_, mixed venous oxygen saturation; TAPSE, tricuspid annular plane systolic excursion; WU, Wood unit.

## Data Availability

Original data include confidential patient information but may be available on reasonable request to the corresponding author.
