# Supplementary material for: Preoperative Right Ventricle–to–Pulmonary Artery Coupling Correlates With Intensive Care Length of Stay After Pulmonary Endarterectomy
Source: J Cardiothorac Vasc Anesth. Author manuscript; Available in PMC 2026 Jun 28. (PMC13310418; doi:10.1053/j.jvca.2025.12.013)
Supplement: 2 [file NIHMS2188397-supplement-2.docx]

**Supplementary Table 1** – Demographics, risk factors, hemodynamics, echocardiographic measures and outcomes stratified by TAPSE/PASP median

| **Variable** | **Level** | **TAPSE/PASP below median** | **TAPSE/PASP above median** | **p-value** |
| --- | --- | --- | --- | --- |
| N |  | 30 | 30 |  |
| TAPSE/PASP, mm/mmHg, |  | .15 (.13, .17) | .29 (.25, .44) | <0.001 |
| Age, years |  | 63 (12) | 56 (18) | 0.10 |
| Sex | Female | 10 (33%) | 15 (50%) | 0.29 |
| BMI |  | 26.6 (25.2, 28.7) | 27.5 (26.3, 33.6) | 0.17 |
| Smoking | Never | 11 (37%) | 15 (50%) | 0.54 |
|  | Former | 17 (57%) | 12 (40%) |  |
|  | Active | 2 (7%) | 3 (10%) |  |
| Pulmonary disease | Yes | 11 (37%) | 4 (13%) | 0.072 |
|  | Asthma | 3 (10%) | 1 (3%) | 0.61 |
|  | COPD | 4 (13%) | 2 (7%) | 0.67 |
|  | Emphysema | 1 (3%) | 0 (0%) | 1.00 |
|  | Fibrosis | 2 (7%) | 0 (0%) | 0.49 |
|  | Sarcoidosis | 2 (7%) | 0 (0%) | 0.49 |
|  | Other | 1 (3%) | 1 (3%) | 1.00 |
| Ischemic heart disease | Yes | 4 (13%) | 2 (7%) | 0.39 |
| Congestive heart disease | No | 30 (100%) | 30 (100%) |  |
| Hjerteklapsygdom | Yes | 5 (17%) | 5 (17%) | 1.00 |
| Stroke/TCI | Yes | 4 (13%) | 0 (0%) | 0.038 |
| Trombofili | Yes | 5 (17%) | 5 (17%) | 1.00 |
| Previous PE | Yes | 28 (93%) | 27 (90%) | 0.64 |
|  |  |  |  |  |
| 6 MWD, meters |  | 360 (295, 480) | 408 (345, 460) | 0.39 |
| Borg skale (CR10) |  | 6.5 (4, 7.5) | 5 (3, 7) | 0.32 |
| NYHA classification |  | 2 (2, 2) | 2 (2, 2) | 0.66 |
| NYHA classification | I | 0 (0%) | 1 (3%) | 0.34 |
|  | II | 6 (21%) | 5 (17%) |  |
|  | III | 20 (71%) | 24 (80%) |  |
|  | IV | 2 (7%) | 0 (0%) |  |
| NT-proBNP |  | 3463.5 (1613, 4600) | 438.5 (139, 848) | <0.001 |
| Creatinine |  | 99 (80, 110) | 82.5 (72, 95) | 0.010 |
| EuroSCORE II |  | 1.605 (1.23, 2.52) | 1.18 (1, 2.11) | 0.064 |
| **Right heart catherization** | | | | |
| mRAP, mmHg |  | 10 (7, 15) | 7 (5, 10) | 0.038 |
| Pulmonary artery systolic pressure, mmHg |  | 89.5 (75, 95) | 77 (58, 88) | 0.005 |
| Pulmonary artery diastolic pressure, mmHg |  | 32 (25, 37) | 25.5 (20, 33) | 0.077 |
| Pulmonary artery mean pressure (mPAP), mmHg |  | 49.5 (46, 56) | 45 (36, 52) | 0.030 |
| Pulmonary Artery Wedge Pressure (PAWP), mmHg |  | 9 (8, 13) | 10.5 (9, 14) | 0.19 |
| Cardiac Output, L/min |  | 3.75 (3.1, 4.4) | 4.85 (4, 5.4) | <0.001 |
| Cardiac Index, L/min/m2 |  | 1.85 (1.6, 2.1) | 2.3 (2.1, 2.7) | <0.001 |
| PVR, WU |  | 10.6 (8.9, 14.1) | 6.3 (4.7, 9.6) | <0.001 |
| SVO2, % |  | 54 (51, 57) | 63 (57, 68) | <0.001 |
| Arterial saturation, % |  | 90 (86, 93) | 91 (88, 95) | 0.25 |
| **Echocardiography** | | | | |
| RA areal, cm2 |  | 27.6 (23.3, 33.4) | 21.6 (18, 25.9) | 0.003 |
| RV diameter, mm |  | 53 (49, 59) | 46 (42, 56) | 0.014 |
| RV/LV Ratio |  | 1.44 (1.15, 1.57) | 1.02 (.88, 1.20) | <0.001 |
| TAPSE, mm |  | 13.75 (11.33, 17.67) | 19.67 (18, 24) | <0.001 |
| PASP on echo, mmHg |  | 94 (85, 104) | 68 (48, 76) | <0.001 |
| TAPSE/PASP, mm/mmHg, median |  | .15 (.13, .17) | .29 (.25, .44) | <0.001 |
| LV ejection fraction, % |  | 62 (60, 66) | 58 (55, 60) | 0.048 |
| **Clinical outcomes** | | | | |
| ICU LOS, hours |  | 90 (50, 136) | 63 (40, 94) | 0.026 |
| Duration of vasopressor in ICU, hours |  | 46.5 (25, 122) | 28 (21, 47) | 0.018 |
| Duration of intubation, hours |  | 29 (27, 51) | 29 (25, 33) | 0.18 |
| Duration of inhaled nitric oxide in ICU, hours |  | 25.5 (17, 32) | 17 (16, 41) | 0.79 |
| Vasoactive-inotropic scale |  | 11.495 (6, 29) | 7 (4.995, 14) | 0.14 |
| Norepinephine equivalent accumulated, ug |  | 6052 (2404, 19976) | 5216 (2308, 12910) | 0.43 |
| Accumulated dobutamine, ug |  | 282.6 (89.7, 656) | 117.3 (11.28, 245.6) | 0.052 |
| Hospital length of stay, days |  | 10 (9, 14) | 9 (7, 14) | 0.22 |

Characteristics of included patients, n=60, stratified by TAPSE/PASP median. Data are presented as median [IQR], mean (SD) or n (%) as appropriate.

Abbreviations: BMI, body mass index; COPD, chronic obstructive pulmonary disease; 6MWD, six minutes walking distance; NT-proBNP, N-terminal pro-brain natriuretic peptide; PVR, pulmonary vascular resistance; WU, Wood Unit; SvO_2_, mixed venous oxygen saturation; RA, right atrial; RV, right ventricle; LV, left ventricle; TAPSE, tricuspid annular plane systolic excursion; PASP, pulmonary artery systolic pressure; ICU LOS, intensive care unit length of stay.
