# Supplementary material for: Preoperative Right Ventricle–to–Pulmonary Artery Coupling Correlates With Intensive Care Length of Stay After Pulmonary Endarterectomy
Source: J Cardiothorac Vasc Anesth. Author manuscript; Available in PMC 2026 Jun 28. (PMC13310418; doi:10.1053/j.jvca.2025.12.013)
Supplement: 3 [file NIHMS2188397-supplement-3.docx]

**Supplementary Table 2**

|  |  | **ICU LOS < median** | **ICU LOS > median** | **p-value** |
| --- | --- | --- | --- | --- |
| N |  | 31 | 29 |  |
| ICU LOS, hours |  | 44 (38, 63) | 135 (94, 187) | **<0.001** |
| Patients characteristics | | | | |
| Age, y |  | 57 (17) | 63 (13) | 0.13 |
| Sex | Female | 12 (39%) | 13 (45%) | 0.79 |
| BMI |  | 26.9 (24.2, 30.6) | 27.4 (26.4, 33.2) | 0.16 |
| Smoking | Never | 13 (42%) | 13 (45%) | 0.80 |
|  | Previous | 16 (52%) | 13 (45%) |  |
|  | Active | 2 (6%) | 3 (10%) |  |
| Pulmonary disease | No | 27 (87%) | 18 (62%) | **0.037** |
|  | Yes | 4 (13%) | 11 (38%) |  |
|  | Asthma | 2 (6%) | 2 (7%) | 1.00 |
|  | COPD | 1 (3%) | 5 (17%) | 0.098 |
|  | Emphysema | 0 (0%) | 1 (3%) | 0.48 |
|  | Fibrosis | 0 (0%) | 2 (7%) | 0.23 |
|  | Sarcoidosis | 0 (0%) | 2 (7%) | 0.23 |
|  | Other | 1 (3%) | 1 (3%) | 1.00 |
| Ischemic heart disease | No | 28 (90%) | 26 (90%) | 0.93 |
| Congestive heart failure | No | 31 (100%) | 29 (100%) |  |
| Heart valve disease | No | 26 (84%) | 24 (83%) | 0.91 |
| Previous pulmonary embolism | No | 3 (10%) | 2 (7%) | 0.70 |
| 6 minutes walking distance, m |  | 425 (340, 540) | 360 (270, 430) | **0.049** |
| Borg scale (CR10) |  | 5 (3, 7) | 5 (5, 7) | 0.54 |
| NYHA class |  | 2 (1, 2) | 2 (2, 2) | **0.031** |
|  | I | 1 (3%) | 0 (0%) | 0.15 |
|  | II | 8 (27%) | 3 (11%) |  |
|  | III | 21 (70%) | 23 (82%) |  |
|  | IV | 0 (0%) | 2 (7%) |  |
| NT-proBNP, ng/L |  | 587 (285, 3500) | 1613 (744, 4142) | 0.085 |
| Creatinine, umol/L |  | 89 (77, 105) | 87 (79, 101) | 0.77 |
| EuroSCORE II |  | 1.19 (1.00, 1.85) | 1.89 (1.35, 2.50) | **0.020** |
| Pre-operative right heart catheterization | | | | |
| Mean right atrial pressure, mmHg |  | 7 (5, 10) | 10 (7, 14) | **0.040** |
| Pulmonary artery systolic pressure, mmHg |  | 76 (59, 92) | 86 (75, 94) | **0.047** |
| Pulmonary artery diastolic pressure, mmHg |  | 24 (17, 33) | 32 (28, 37) | **0.002** |
| Pulmonary artery mean pressure, mmHg |  | 45 (36, 51) | 51 (46, 57) | **0.006** |
| Pulmonary artery wedge pressure, mmHg |  | 10 (7, 13) | 10 (8, 13) | 0.54 |
| Cardiac Output, L/min |  | 4.4 (3.8, 5) | 3.9 (3.3, 5.1) | 0.27 |
| Cardiac Index, L/min/m^2^ |  | 2.1 (1.8, 2.5) | 2.1 (1.6, 2.3) | 0.32 |
| PVR, WU |  | 7.9 (5.4, 10.5) | 10 (6.8, 14.1) | **0.026** |
| SvO_2_, % |  | 59 (55, 66) | 55 (51.5, 62) | 0.090 |
| Arterial saturation, % |  | 92 (90, 95) | 89 (86, 93) | **0.022** |
| Pre-operative transthoracic echocardiography | | | | |
| RA area, cm^2^ |  | 23.8 (18.9, 27.6) | 24.3 (19.6, 33.6) | 0.32 |
| RV diameter, mm |  | 50 (44, 56) | 52 (45, 60) | 0.24 |
| RV/LV ratio |  | 1.09 (0.89, 1.36) | 1.31 (1.01, 1.50) | 0.080 |
| TAPSE, mm |  | 18.7 (13.7, 20.7) | 17.3 (13.3, 19.7) | 0.46 |
| TAPSE/PASP, mm/mmHg |  | 0.22 (0.17, 0.32) | 0.17 (0.15, 0.25) | 0.13 |
| LV ejection fraction, % |  | 60 (55, 61) | 61 (57, 66) | 0.33 |
| Vasopressor need | | | | |
| Accumulated norepinephrine equivalence, ug |  | 4792 (2454, 7496) | 16752 (2272, 42472) | **0.029** |
| Accumulated dobutamine, ug |  | 127 (12, 242) | 385 (14, 716) | **0.031** |

Caption: Characteristics of included patients, n=60. Data are presented as mean (SD), median [IQR], or n (%) as appropriate. Abbreviations: BMI, body mass index; COPD, chronic obstructive pulmonary disease; 6MWD, six minutes walking distance; NYHA, New York Heart Association; NT-proBNP, N-terminal pro-brain natriuretic peptide; PVR, pulmonary vascular resistance; WU, Wood Unit; SvO_2_, mixed venous oxygen saturation; RA, right atrial; RV, right ventricle; LV, left ventricle; TAPSE, tricuspid annular plane systolic excursion; PASP, pulmonary artery systolic pressure; ICU LOS, intensive care unit length of stay.
